# Green Synthesis and Antibacterial Activity of HAp@Ag Nanocomposite Using *Centella asiatica* (L.) Urban Extract and Eggshell

**DOI:** 10.1155/2020/8841221

**Published:** 2020-10-01

**Authors:** Xuan Nui Pham, Hoa Thi Nguyen, Ngan Thi Pham

**Affiliations:** Department of Chemical Engineering, Hanoi University of Mining and Geology, 18 Vien Street, Duc Thang, Bac Tu Liem, Hanoi, Vietnam

## Abstract

In recent years, the green synthesis of nanoparticles via biological processes has attracted considerable attention. Herein, we introduce a facile and green approach for the synthesis of poriferous silver nanoparticles (Ag-NPs) decorated hydroxylapatite (HAp@Ag) nanoparticles with excellent antibacterial properties. All the nanocomposites were fully characterized in the solid state via various techniques such as X-ray powder diffraction (XRD), Fourier transform infrared spectroscopy (FT-IR), scanning electron microscopy (SEM), and energy-dispersive X-ray spectrometer (EDX), in which the synthesized Ag-NPs (24 nm in diameter) and their homogeneous incorporation on HAp have been studied by ultraviolet-visible (UV-vis) technique, transmission electron microscopy (TEM), and dynamic light scattering (DLS) analysis. The obtained results indicate that the structure and morphology of HAp have no significant changes after the incorporation of Ag-NPs on its surface. Moreover, an impressive antibacterial activity of HAp@Ag nanocomposite against Gram-positive bacterium *Staphylococcus aureus* and Gram-negative bacteria *Escherichia coli* and *Pseudomonas aeruginosa* has been recorded by using the agar well diffusion method. As a result, the HAp@Ag nanocomposite promises to be a great biomedical material with high antibacterial properties.

## 1. Introduction

Silver nanoparticles (Ag-NPs) have been widely investigated owing to their great antibacterial ability, even at low concentrations [1–4]. Various techniques have been proposed for the synthesis of Ag-NPs, consisting of physical methods, mechanical methods such as grinding and deformation, and chemical methods. Unfortunately, such methods face challenges arising from their toxicity and the manufacturing cost. Green synthetic techniques have been proposed to overcome these problems due to their positive influence on the environment and ability to produce a large number of high-purity NPs [5–8]. These techniques utilize plant extracts, which play a role as effective reducing and stabilizing agents for salt ions to form uniform nanoparticles [9, 10]. Recent research has demonstrated that the synthetic process of Ag-NPs using leaf extracts such as *Polyalthia longifolia, Geranium (Pelargonium graveolens), Eucalyptus citriodora (neelagiri), Ficus benghalensis (marri),* and *Penicillium oxalicum* can produce NPs with average size from 16 to 58 nm [11–15].

Accordingly, silver nanoparticles have been synthesized using reishi mushroom (*Ganoderma lucidum*) extract. The results showed that Ag-NPs are spherical with a diameter range of 15–22 nm [16]. Şahin et al. [17] investigated the effect of pomegranate extract and monodisperse silver nanoparticle combination on MCF-7 cell line. According to this study, the silver nanoparticles were synthesized using the pomegranate extract. Gӧl et al. [18] reported the use of green synthesized silver nanoparticles with the aid of *Camellia sinensis* extract to provide antibacterial activity on ceramic structure. *Rhododendron ponticum* extracts were used for the green synthesis of biogenic silver nanoparticles and their effects on antibacterial and anticarcinogenic activity were reported by Korkaz et al. [19]. Aygün et al. [20] reported the antimicrobial and anticarcinogenic properties of silver nanoparticles obtained by green synthesis using the extract of *Rheum ribes*, a medicinal plant. Another study also reported [21] the green synthesis of monodisperse platinum nanoparticles (Pt-NPs) by using pomegranate extract and effectiveness of Pt-NPs was determined by cell viability, propidium iodide staining test, flow cytometry, and comet tests on the MCF-7 cancer cell line.

Hydroxyapatite (HAp) corresponds to the chemical formula Ca_10_(PO_4_)_6_(OH)_2_ or Ca_5_(PO_4_)_3_(OH) with hexagonal crystallites. This structure is usually found in bone and dentin, and the single-crystal structure is also found in dental enamel. HAp has been employed and researched in various fields, particularly, in biomedical engineering for various applications including drug delivery, gene transfer, and biological analysis, owing to its promising characteristics in bioactivity and biological compatibility with cells and tissues. For example, HAp can enhance the osteoblast adhesion to accelerate the reformation process of bone against the rejection of the body [22–26]. HAp can be synthesized using different methods including sol-gel [27], precipitation [28], and hydrothermal [29–31].

Interestingly, in recent years, natural materials such as fishbone, bovine bone, coral, *Ostrea* shell, and eggshell have been used as an important ingredient in the HAp synthetic process. In a new report, Lemos et al. [32] demonstrated the change of natural aragonite from squid bone to form HAp via a heat treatment process at 200°C. Similarly, Ooi et al. [33] and Sivakumar's group [34] described an effective hydrothermal method using bovine bone and coral precursors, respectively, to form porous HAp. Ho et al. [31] have also reported that HAp was successfully synthesized using a hydrothermal process from both eggshell and biological substances from fruit waste extract. By using waste material sources, this will help to save more cost and more safety as well as environmental friendliness. With raw material as the eggshell-waste by-products from daily life, which occupies 11% upon the mass total of an egg and gets major ingredient to be calcium carbonate [35], it is also a promising material source to replace for “chemical apatite” using in simple and valuable synthetic processes.

There are obvious benefits and promising characteristics of HAp in using to replace human bone and teeth. However, it must be stressed that, during the process of transplant surgery, many important problems related to bone rejection, osteomyelitis, and swelling as well as the fracture should be still solved. Additionally, osteomyelitis is also a direct cause affecting the success of the surgery. Therefore, the fabrication and development of new materials, which not only get highly biocompatible, replaceable, and orthopedic but also antibacterial ability, are an extremely important and necessary research direction with the development of biochemical material at present. There are several researches involving in HAp@Ag nanocomposite material; for example, Lee et al. [36] investigated the synthetic process of calcium phosphate-Ag membrane in medical applications; meanwhile, Feng et al. [37] determined the antibacterial ability of Ag-HAp membrane on alumina substrate, and Marsick et al. [38] demonstrated promising properties of Ag-NPs in nanocomposite materials by bone tissue engineering.

**Figure 1:**
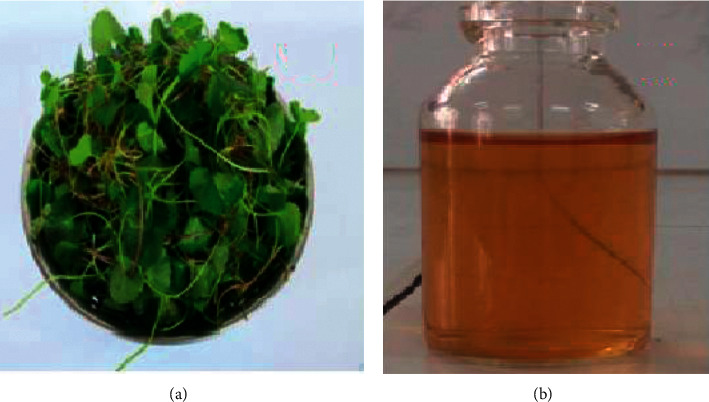
Digital photographs of (a) *Centella asiatica* (L.) Urban and (b) *Centella asiatica* (L.) Urban extract.

In this work, HAp@Ag nanocomposite has been synthesized to maximize all advantages of HAp material and the excellent antimicrobial ability of Ag-NPs. Ag-NPs have been synthesized by *Centella asiatica* (L.) Urban extract (Figure 1), while eggshell has been used for the synthetic process of HAp. On the basis of abundant precursors, it brings out many advantages over other methods such as simplicity and low cost, but the synthetic performance is still high. The synthesized materials have been characterized by different techniques as well as testing the antibacterial activity.

## 2. Experimental

### 2.1. Materials

Eggshells and *Centella asiatica* (L.) Urban leaves were collected from local sources. Silver nitrate (AgNO_3_, >99.98%) of analytical grade was purchased from Sigma-Aldrich. Acid phosphoric (H_3_PO_4_, 85%), ammonia solution (NH_3_, 28%), and ethanol (C_2_H_5_OH, >98%) were purchased from Sinopharm Chemical Reagent Co. Ltd., China. All aqueous solutions were prepared using distilled deionized (DI) water.

### 2.2. Synthesis of Silver Nanoparticles (Ag-NPs) from *Centella asiatica* (L.) Urban Leaf

Firstly, the *Centella asiatica* (L.) Urban leaves were carefully washed with DI-water to remove impurity and then dried for 2 days at room temperature. 100 g of dried leaves was milled by the ordinary coffee grinder and extracted by the Soxhlet system with a 80 : 20 (v/v) ratio of DI-water and ethanol. After 4 h, the extract was separated and evaporated to completely remove ethanol. The final extract was collected for further usage.

For the synthesis of Ag-NPs, a suitable aqueous solution of *Centella asiatica* (L.) Urban extract was gradually added into the mixture including 20 mL of 0.01 M silver nitrate (AgNO_3_) solution in an Erlenmeyer flask on the magnetic stirrer at 40°C with 300 rpm for 60 min. Then the whole flask was kept in dark place at room temperature for 24 h. The color of the solution changed from the yellowish-brown to the dark-brown after 30 min of the heating process. The mixture changes a color of extract solution from the yellow-brown to the black-brown which represents the reduction of Ag^+^ to Ag^o^ by the reducing agents in the extract. Next, the above mixture was continuously stirred at different times from 60 to 240 min. The Ag-NPs were separated by centrifugation at 4°C with 12,000 rpm using high speed refrigerated centrifuge, and finally, the obtained Ag-NPs solution was stored at 4°C and analyzed by UV-vis spectroscopy.

### 2.3. Synthesis of Hydroxyapatite (HAp)

Eggshell powders were used as precursor for the preparation of HAp. Eggshells were cleaned with DI-water and boiled to remove odors and impurities. After preliminary treatment, the eggshells were ground into a fine powder and calcined at 300°C to remove organic ingredients and then calcined a second time at 900°C to decompose CaCO_3_ into CaO. The obtained product was in the form of white fine powder.

White fine powder containing CaO was then fully dissolved in 100 mL of DI-water and stirred within 30 min at room temperature to obtain a mixture with the desired concentration of CaO (solution A). After that, 0.3 M aqueous solution of H_3_PO_4_ was added dropwise to solution A with constant stirring. The molar ratio of Ca^2+^/PO_4_^3−^ was kept at 1.7 and aqueous ammonia solution was employed to adjust pH to 10; the resulting mixture was continuously stirred for 4 h while fixing the investigated temperature and stirring speed during reaction time. The final product was created as white suspension form and was stabilized for 72 h. In the next step, this mixture was filtered and dried at 60°C for 12 h. Finally, the dried powders were calcined at 500°C, 900°C, and 1100°C for 4 h.

### 2.4. Synthesis of Silver Nanoparticles-Hydroxyapatite (HAp@Ag) Nanocomposite

The HAp@Ag nanocomposite was synthesized by incorporating Ag-NPs on HAp surface via the embedding method. 100 mg of HAp powder was dispersed in 100 mL of Ag-NPs solution. The mixture was continuously stirred for 2 h at room temperature. The resulting slurry of the HAp@Ag nanocomposite was kept overnight at room temperature. Finally, the HAp@Ag nanocomposite was extensively washed with DI-water, filtered, and then dried at 60°C for 12 h, yielding the HAp@Ag powder with a brown color.

### 2.5. Characterization

X-ray diffraction (XRD) patterns of the samples were acquired with a Bruker-Germany powder diffractometer equipped with a CuK*α* radiation source (*λ* = 1.541 Å) within the range of 2*θ* = 10–70° at a scanning rate of 5°/min and a step time of 1 s. The morphology and structure of the synthesized samples were investigated using scanning electron microscopy (SEM, Hitachi S-4800, Japan), equipped with an energy-dispersive X-ray spectrometer (EDX) and transmission electron microscopy (TEM, Leica IEO 906E) operating at 120 kV. Fourier transform infrared spectra (FT-IR, Prestige-21, Shimadzu) were examined on KBr pellets techniques, working in the range of wavenumber 4000–400 cm^−1^. UV-vis spectroscopic studies were carried out using a UV-2450 double-beam spectrophotometer (Shimadzu, Tokyo, Japan), which was operated in the range of 200–800 nm. The average size and size distribution of the Ag-NPs were measured by dynamic light scattering (DLS, Malvern Instruments Ltd., UK) Nano Zetasizer.

### 2.6. Antibacterial Activity of HAp@Ag Nanocomposite

Using the agar well diffusion method [39], the antibacterial activity of the HAp@Ag nanocomposite was tested against pathogenic bacterial species *S. aureus* (ATCC 13709) as a model of Gram-positive bacteria that are commonly found through a break in the skin. The two microorganisms such as *E. Coli* (ATCC 25922) and *P. aeruginosa* (ATCC 15442) were used as models for Gram-negative bacteria. These strains of bacteria were cultured from the tube of original strains in LB (Luria–Bertani) environments at 37°C, incubated overnight. These bacteria were obtained from the laboratory of the superstructure, Virus Department, National Institute of Hygiene and Epidemiology, Hanoi, Vietnam.

The activity test plate is prepared by inoculating a bacterial solution of 200 *µ*L, equivalent to 10^7^ CFU/mL onto the surface of the Petri dish containing a solid LB solution, allowing it to dry and chisel from 5 to 6 wells. The tested HAp@Ag sample was solubilized with dimethyl sulfoxide (DMSO) and Ag-NPs aqueous extract of the diluted sample solution in sterile water (100 mg/mL). 50 *µ*L of the test solution was added to the agar wells on Petri dishes and the dishes were kept at room temperature for 2 h, until the test solution from the wells diffuses to the bacterial culture environment. Then, the dishes are placed in an incubator at 37°C for 24 h. The positive control is an antibiotic solution (ampicillin 0.1 mg/mL with *E. coli* and Kanamycin 5 mg/mL with *S. aureus*), and the negative control is DMSO. The size of the inhibition zones corresponded to D-d (mm) where D is the inhibition ring diameter (mm) and d is the diameter of agar (mm). The experiment was repeated three times and the mean radius value was taken.

## 3. Results and Discussion

### 3.1. Synthesis and Characterization of Ag-NPs Using *Centella asiatica* (L.) Urban Extract

**Figure 2:**
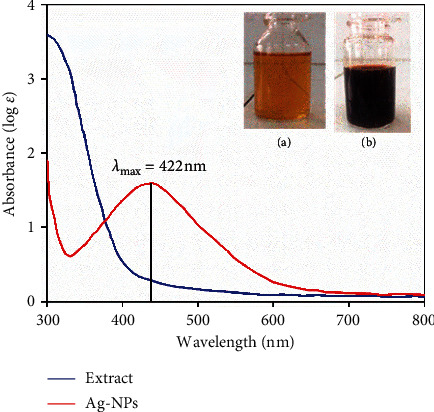
UV-vis absorption spectra of Ag-NPs and *Centella asiatica* (L.) Urban leaf extract. Inset: (a) extract and (b) silver nanoparticles.

The silver ions were reduced to form Ag-NPs during the interaction process with *Centella asiatica* (L.) Urban extract. The results of UV-vis spectra show a strong surface plasmon resonance (SPR) band at 422 nm, confirming the formation of Ag-NPs in the aqueous extract as shown in Figure 2.

**Figure 3:**
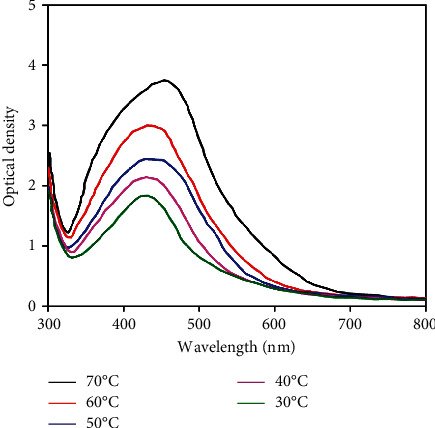
UV-vis absorption spectra of silver nanoparticles (Ag-NPs) recorded as a function of reaction temperature of 0.01 M AgNO_3_ solution with leaf extract. Reaction time: 120 min; extract volume: 7 mL.

Figure 3 shows the effect of reaction temperature of 0.01 M AgNO_3_ solution with leaf extract on formation of silver nanoparticles. The results showed that when the reaction temperature was elevated from 50 to 70°C, the SPR band shifted from 422 to 435 nm in addition to the simultaneous broadening of the band. It can be seen that concentration of silver nanoparticles in aqueous colloidal solution reached the highest at 70°C. Along with that is the increase in particle size SPR band broadened. Meanwhile, silver nanoparticles concentration increased mildly with temperature from 30 to 50°C and the wavelength of the strong absorption did not shift during the reaction. Balancing concentration and size of silver nanoparticles and reaction temperature was optimized at 50°C.

**Figure 4:**
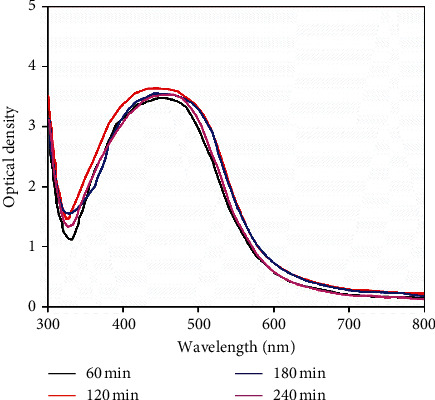
UV-vis absorption spectra of silver nanoparticles recorded as a function of reaction time of 0.01 M AgNO_3_ solution with leaf extract. Reaction temperature: 50°C; extraction volume: 7 mL.

Figure 4 shows the effect of reaction time on the formation of Ag-NPs. As can be seen, the presence of the strong absorption peak represented the existence of Ag-NPs, in which the position of major peak was located at 422 nm. In particular, the peak intensity was the highest after reaction for 120 min, which may explain the idea that increasing reaction time causes significant aggregation of the Ag-NPs resulting in size increase, thus the change of morphology as well as the reduction of the energy emission and surface plasmon resonance [40].

**Figure 5:**
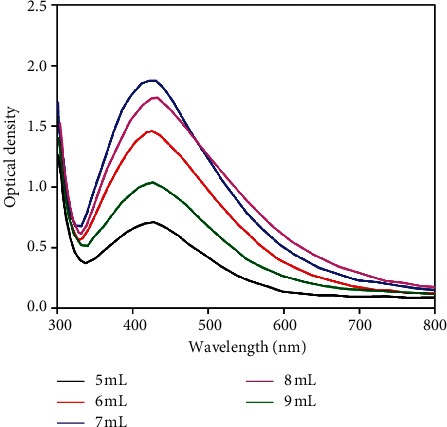
UV-vis absorption spectra of silver nanoparticles recorded as a function of 0.01 M AgNO_3_ solution with different extract volumes. Reaction temperature: 50°C; reaction time: 120 min.

Similarly, according to the UV-vis result (Figure 5) of silver nanoparticles solution at the different extract volumes, the strongest absorption peaks of Ag-NPs were found at *λ*_max_ = 422 nm. Increasing the extract volume from 5 to 7 mL increased mildly the absorption intensity. However, if the extract volume increased up to 8-9 mL, the absorption intensity tended to decrease gradually. Theoretically, when increasing the extract volume, the reaction will happen faster and the amount of Ag-NPs will be created more in a short time. At this time, PVA as an important stabilizer could not help protect all formed Ag-NPs from aggregation or the fast growth of these crystals. So, the obtained Ag-NPs having large and nonuniform sizes lead to decrease in the absorbability of UV-vis.

**Figure 6:**
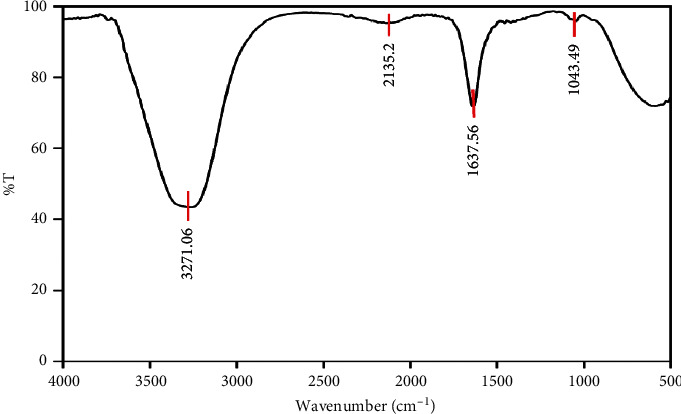
FT-IR spectrum of raw *Centella asiatica* (L.) Urban extract.

FT-IR spectroscopy was carried out to identify the different functional groups of *Centella asiatica* (L.) Urban extract.  Figure 6 indicates the presence of four bands at 3271, 2135, 1637, and 1043 cm^−1^. The strong absorptions at 3271 cm^−1^ and 2135 cm^−1^ were assigned to secondary amides (−NH) and the ketene groups (C=C=O), respectively [41]. The band around 1637 cm^−1^ indicates that primary and secondary amides exist in the extract. The band at 1043 cm^−1^ revealed the presence of a C–O stretching vibration. This band could be used to assign a primary, secondary, or tertiary structure of alcohol or to confirm the presence of phenolic compounds. The obtained results demonstrated that these functional groups played important roles in the reduction of Ag^+^ ions to create Ag-NPs that are in agreement with Ling et al. [42] and Mondal et al. [43] in the literatures.

**Figure 7:**
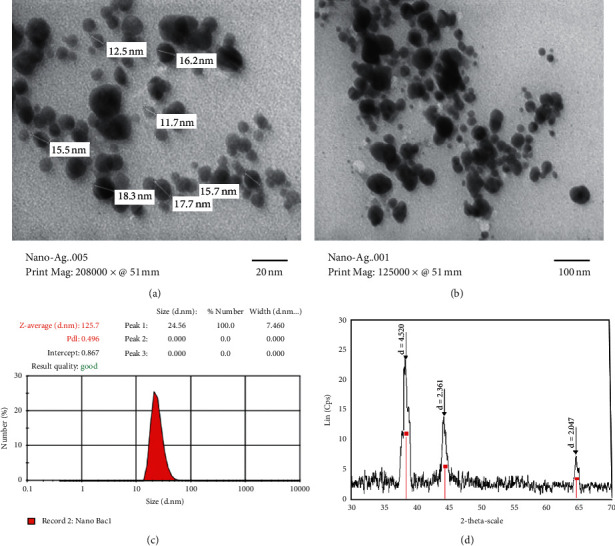
(a, b) TEM images, (c) size distribution by zeta potential measurement, and (d) XRD patterns of silver nanoparticles synthesized from 0.01 M AgNO_3_ solution and 7 mL of *Centella asiatica* (L.) Urban extract.

TEM images as well as particle size distributions and XRD patterns of Ag-NPs are shown in Figure 7. The micrographs show that Ag-NPs, in spherical form, were uniformly distributed throughout the solution without aggregation (Figures 7(a) and 7(b)). The particle size distribution indicates an average particle diameter of 15–60 nm (Figure 7(c)). The XRD patterns of Ag-NPs in Figure 7(d) show reflection peaks at 2*θ* values of 38.11°, 44.52°, and 64.43°, corresponding to the reflection faces (110), (200), and (220), which can be assigned to the face-centered cubic (fcc) lattice silver (JCPDS no. 99-0094). This was once again confirmed by the formation of Ag-NPs in the reaction solution with the presence of interaction between Ag^+^ ions and other ingredients in the plant extract. Using Debye–Scherrer's equation for calculation, the crystalline size of NPs is calculated at approximately 27 nm (*λ* = 1, 54 Å, *β* = 0.00492 rad, 2*θ* = 38°).

Some researchers have used the extracts of plants for synthesis of the Ag-NPs [5, 44–48]. The results show that silver nanoparticles were synthesized from extracts of various plants in the size range of 2–100 nm. In our research, the Ag-NPs synthesized from the *Centella asiatica* (L.) Urban leaf extract have sizes ranging from 20 to 60 nm, and the average particle size is about 24.56 nm. The sizes of the Ag-NPs formed from extracts of various plants are listed in Table 1.

### 3.2. Synthesis and Characterization of Hydroxyapatite (HAp) Using Eggshell

**Figure 8:**
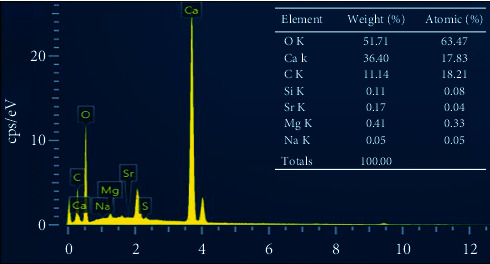
EDX spectrum of the eggshell after calcination process.

Figure 8 shows the EDX pattern of the treated eggshell sample after the calcination process. The EDX pattern indicates the presence of constituents such as O, Ca, C, Si, Sr, Mg, and Na in the eggshell sample. In particular, Ca, C, and O are main components in CaCO_3_ and CaO with 99.25 wt.% of the total content of mineral elements while the others were only found in trace (0.75 wt.%) that had no effect on the crystalline structure of as-prepared HAp, and its main composition is compatible with human bone [34].

**Figure 9:**
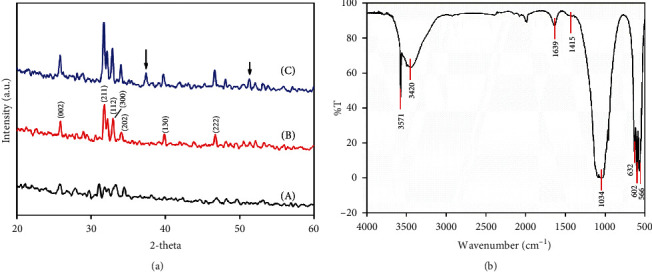
(a) X-ray diffraction patterns of hydroxyapatite powders calcined at different temperatures: (A) 500°C; (B) 900°C; (C) 1100°C. (b) FT-IR spectrum of hydroxyapatite powders calcined at 900°C.

The phase structure of the HAp sample was identified by powder X-ray diffraction.  Figure 9(a) shows the XRD patterns of the synthesized HAp from eggshell (a, b, and c) at different calcination temperatures of 500°C, 900°C, and 1100°C, respectively. It can be clearly seen that the main phase, as expected, was hydroxyapatite, which has the highest peak at a 2*θ* value of 31.77°, corresponding to the (211) lattice plane of crystalline hydroxyapatite (JCPDS 72-1243). Besides, the XRD pattern also showed the presence of other peaks at 2*θ* values of 25.90, 33.01, 34.04, 39.70, 46.68, 49.50, and 53.10°, which can be assigned to diffraction of the (002), (300), (202), (310), (222), (213), and (004) lattice planes in the apatite phase of the HAp structure. In the case of sample calcined at 500°C (Figure 9(a)), the XRD pattern showed the presence of an amorphous phase. In contrast, at a calcination temperature of 900°C, the sharpness of characteristic peaks was clearly enhanced (Figure 9(a)). Similarly, for the sample calcined at 1100°C, the formation of a secondary phase (CaO) was identified, corresponding to diffraction peaks at 37.37 and 53.86° (2*θ*).

FT-IR analysis was performed to determine the existence of surface functional groups in HAp. In Figure 9(b), there are two strong absorption bands at 566 and 602 cm^−1^, which can be attributed to asymmetric and symmetric stretching vibrations modes (*υ*_4_) in the O-P-O of the phosphate group. A strong absorption band at 1034 cm^−1^ was ascribed to a characteristic band asymmetric stretching mode (*υ*_3_) of PO_4_^3−^ group. The broad bands in the wavenumber range of 3420 cm^−1^ belong to traces of hydroxyl in water molecules that are incorporated in the HAp structure and a weak band at 1639 cm^−1^, which suggests that the resolved peaks are due to O-H stretching of water in the high energy region. This also indicates that the intermolecular hydrogen bonding of water molecules is not suitable [35, 49]. The weak bands at 632 and 3571 cm^−1^ were assigned to the hydroxyl stretching vibration mode of H-O-H in the crystal lattice [49]. The detected peak at 1415 cm^−1^ was attributed to the vibrational modes of the CO_3_^2−^ group, which has also indicated the carbonate groups substitution at the phosphate ions site (B-type) in the crystal lattice [32, 50].

### 3.3. Characterization and Antibacterial Activity of Silver-Hydroxyapatite (HAp@Ag) Nanocomposite

**Figure 10:**
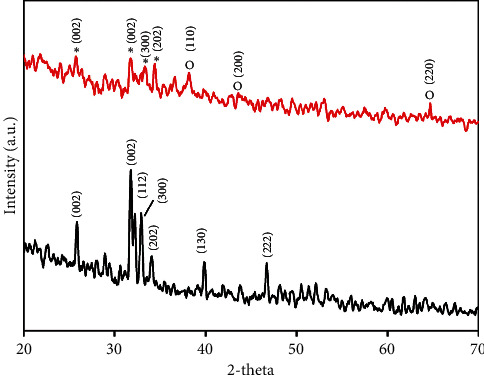
X-ray patterns of (A) HAp and (B) HAp@Ag nanocomposite.

XRD characterization of HAp (Figure 10(a)) and HAp@Ag nanocomposite (Figure 10(b)) showed the specific diffraction pattern of the crystalline hydroxyapatite [HAp, Ca_3_(PO_4_)_3_(OH)]. Furthermore, as shown in Figure 10(b), the sample exposed the minor peaks at 38.3°, 44.1°, and 64.4°, corresponding to the diffractions of the (111), (200), and (200) for the face-centered cubic lattice planes of Ag crystal (JCPDS no. 04-0783).

**Figure 11:**
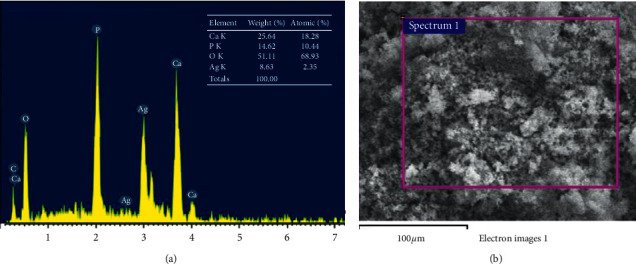
The EDX spectrum of HAp@Ag.

Figure 11 presents the EDX spectrum of the highlighted region containing the quantified elements of the HAp@Ag sample. The spectrum confirms the presence of Ca, P, O, and Ag elements with a Ca/P ratio of 1.75, which is in accordance with the theoretical ratio of hydroxyapatite (Ca/P = 1.67) [51].

**Figure 12:**
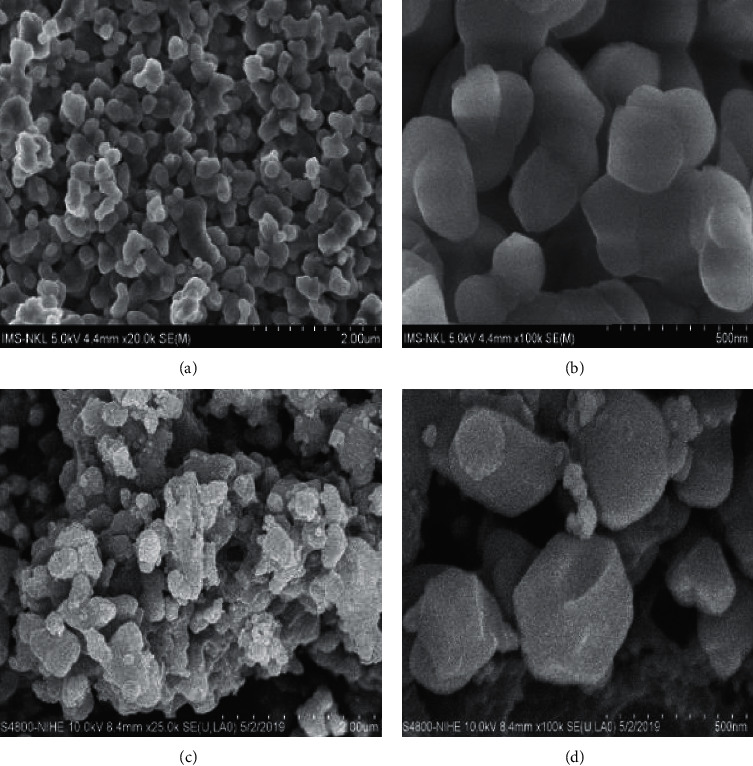
(a, b) SEM image of (hydroxyapatite) HAp and (c, d) HAp@Ag nanocomposite.

SEM images of pure HAp and HAp@Ag nanocomposite are shown in Figure 12. The micrographs of HAp exhibited an ellipsoidal morphology of 100–150 nm in size (Figures 12(a) and 12(b)). In comparison with the pure HAp, it was found in Figures 12(c) and 12(d) that the structure and morphology were retained in the HAp@Ag nanocomposite, although HAp surface was incorporated with Ag-NPs of 24 nm in sizes.

### 3.4. The Antibacterial Activity of HAp@Ag Nanocomposite

The antibacterial activity is evaluated based on the size and diameter of inhibition zones on the agar plate. The size of the inhibition zones corresponded to D-d (mm) where D is the inhibition ring diameter (mm) and d is the diameter of agar (mm). Antibacterial properties are expressed when the antibacterial ring diameter is larger than 2 mm [52]. Where the antibacterial ring is less than 5 mm corresponds to weak resistance, in the range of 5–10 mm to average resistance, and larger than 10 mm to strong resistance.

Table 2 presents the antibacterial activity of the Ag-NPs and HAp@Ag nanocomposite against *E. Coli, S. aureus*, and *P. aeruginosa* by using the agar well diffusion method.

As shown in Table 2, the Ag-NPs and HAp@Ag nanocomposite were effective against all tested microorganisms. Particularly, the inhibitory zone recorded with Gram-positive *S. aureus* bacteria reached 17.0 mm and 16 mm inhibitory zone, respectively. In the case of Gram-negative *E. coli* and *P. aeruginosa*, the inhibitory zone reached 10 mm and 20 mm, respectively. This indicated significant antibacterial activity of the HAp@Ag nanocomposite for both bacterial strains. Furthermore, Ag-NPs showed a higher ability to repress the growth of the microorganism than the HAp@Ag nanocomposite. Indeed, diameter zone of inhibition of Ag-NPs against *S. aureus* was 18 mm, followed by *C. albicans* with 17 mm, then 23 mm for *E. coli*, and 20 mm for *P. aeruginosa*.

**Figure 13:**
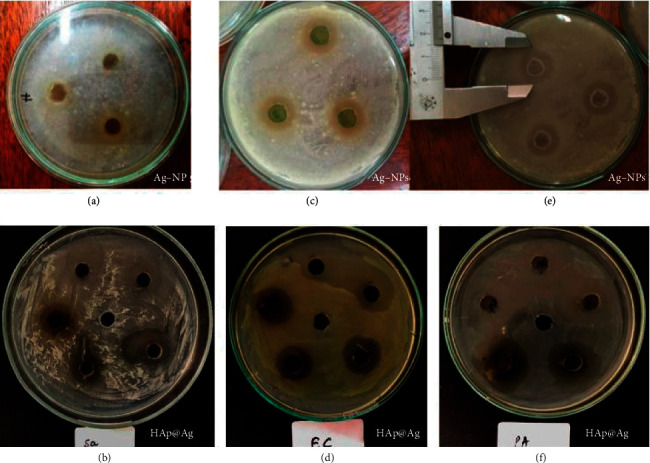
Inhibition zone photographs of the Ag-NPs and HAp@Ag nanocomposites against bacteria *S. aureus* (a, b), *E. coli* (c, d), and *P. aeruginosa* (e, f). The positions (a, c, e) correspond to Ag-NPs concentration of 120 ppm, and the positions (b, d, f) correspond to HAp@Ag nanocomposite concentration of 1.25 mg·mL^−1^.

For comparison, Maliszewska et al. [53] synthesized Ag-NPs using wild strains of *Penicillium* isolated from the environment. The obtained Ag-NPs were evaluated for their antibacterial activity against *B. cereus, S. aureus*, *E. coli*, and *P. aeruginosa* and exhibited inhibitory zones of 12.0–16.0 mm. Similarly, Mohammed et al. [40] used an aqueous extract of *E. camaldulensis* leaves for the synthesis of Ag-NPs. The tested results exhibited that the inhibition zone was around 9.0–14.0 mm for both Gram-negative bacteria (*P. aeruginosa* and *E. coli*) and Gram-positive bacteria (*S. aureus* and *B. subtilis*) (Figure 13).

The Ag-NPs were found, in this study, to possess higher antibacterial activity than those in earlier reports [40, 53]. Ag-NPs with *Lysimachia foenum-graecum* Hance extract 0.003 wt.% of Ag show the inhibition zone diameter of 15.58 ± 0.51 mm for *E. coli* [54]. On the one hand, the bactericidal mechanism of Ag-NPs may be demonstrated via the localization of NPs within cell walls and bacterial membranes. It can inhibit the activity of enzymes and promote the rupture of membranes and cell walls. The cytoplasm membrane detached from the cell wall and therefore DNA lost its replication ability [55, 56]. There are various opinions regarding the mechanism for the antibacterial activity of Ag-NPs. Several studies suggested the antibacterial effect of Ag-NPs regarding the formation of pits in the cell wall leading to an increase in permeability of the cell membrane, leaving bacterial cells incapable of properly regulating transport through the plasma membrane, resulting in cell death [57, 58]. Lok et al. [59] revealed that immediate exposure of microorganism cells to the active concentration of nanosilver resulted in instability of the outer membrane and dissipation of proton motive force. The obtained results also show a higher antibacterial effect for Ag-NPs compared to the HAp@Ag nanocomposite under identical experimental conditions. This may be due to the slow release of Ag-NPs from the HAp@Ag nanocomposite, which interacts with proteins and enzymes of bacteria [60].

## 4. Conclusions

In conclusion, the HAp@Ag nanocomposite, with antibacterial activity, was synthesized using *Centella asiatica* (L.) Urban extract and eggshell as precursors. The optimum reaction conditions for the synthesis of Ag-NPs were investigated. High crystallinity of hydroxyapatite was obtained from eggshell used as a raw source material for the fabrication of HAp@Ag. The Ag-NPs were doped onto the HAp surface via the embedding method for the formation of the HAp@Ag nanocomposite. This material was characterized by various techniques. The results showed that Ag-NPs were uniformly decorated on the surface of HAp and the loading of silver did not influence the HAp morphology and structure. The HAp@Ag nanocomposite exhibited excellent antibacterial activity against Gram-positive bacterium *S. aureus* and Gram-negative bacteria *E. coli* and *P. aeruginosa* via agar well diffusion method. Furthermore, the work for the green synthesis of HAp@Ag may provide potential application as bone substitution reconstructive surgery.

## Figures and Tables

**Table 1:** The size of the silver nanoparticles synthesized from extracts of various plants.

Type of extract	Size range (nm)	Average particle diameter (nm)	Refs.
*Eucalyptus* leaf	4–60	23.25	[5]
Tea leaf	2–9	4.06	[44]
Carob leaf	5–40	18	[45]
*Berberis vulgaris* leaf and root aqueous	10–70	50	[46]
*Capparis spinosa* L. leaf	10–40	20	[47]
*Azadirachta indica* aqueous leaf	10–100	34	[48]
*Centella asiatica* (L.) Urban	20–60	24.56	Our work

**Table 2:** Diameter zones of the antimicrobial activity of the HAp@Ag nanocomposite for both bacterial strains.

Tested microorganisms	Inhibition zones (nm)	
	Ag-NPs	HAp@Ag
*S. aureus*	18 ± 1.1	17 ± 1.2
*E. coli*	23 ± 0.8	10 ± 0.9
*P. aeruginosa*	20 ± 0.9	20 ± 0.9

Values of three replicates are expressed as mean ± SD (standard deviation).

## Data Availability

The UV–VIS absorption analysis data used to support the findings of this study are included in Figures 1–4 within the article. The size distribution data used to support the findings of this study are included in Figure 6(c) within the article.
